# Supplementary material for: A mRNA Vaccine for Crimean–Congo Hemorrhagic Fever Virus Expressing Non-Fusion GnGc Using NSm Linker Elicits Unexpected Immune Responses in Mice
Source: Viruses. 2024 Feb 28;16(3):378. doi: 10.3390/v16030378 (PMC10975845; doi:10.3390/v16030378)
Supplement: Supplementary file 1 [file viruses-16-00378-s001.zip › viruses-2783751-supplementary.pdf]

Supplementary materials

N-terminus

vLMn
MAEGGSSDCISRTQLVKTEVAEIHEDSYGGPGEKITICNGSTIVDQRL
GSELGCYTINRVKSYKLCENSASGKSCEIDSIPVKCKQGFCLKITQEGR
GHVKLSRGTEIVLDVCDSSCELMIPKGTGDILVDCSGGQQHFLQNNL
IDLGCPNVPLLGKMAIYVCRMSNHPKTTMAFLFWFSFGYVITCIVCR
VLFYLLIAIGTVGKKLKQYRELKPQTCIVCESVPVNAIDAEMHELNCSY
NICPYCASRLSTDGLIRHVTQCPKRREKVEETELFLNLERIPWLV

C-terminus

N-terminus

vLMc
MFLDSIAKGMKSLLNSTSLETSLSIEAPWGAINVQSTFKPTVSAANIA
LSWSSVEHRGNKILVSGRSESIMKLEERTGVSWNLGVEDASESKTLTV
SVMDLSQMYSPVFEYLSGDRQVEEWPKATCTGDCPERCGCTSSTCL
HKEWPHSRNWRCNPTWCWGVGTGCTCCGLDVKDFFTDYMLVK
WKVEYIKTEAIVCVELTSQERQCSLIEAGTRFNLGPVTITLSEPRNVQQ
RLPPEIVTLHPKIEEGFFDLMHVQKILSASTVCKLQSCTHGVPGDFQV
YHVGNLLRGDKVNGHSIHKIEPHFNTSWMSWDGCDLDYFCNMGD
WPSCTYTGVTQHNRAAFINMLNIETDYTKTFHFHSKRVTAHGDTPQ
LDLKARPAYGAGEVTVLVEVADLELHTKKLEVSGLKIASLTCSGCYACS
SGISCKVRIHVNEPDEFTVHVRSSDPDVVAAGSSLMARKIEFGADSTF
KAFSSMPKDSLCFYIVEKDYCSSCTDDDTQKCVNTKLDHPQSILIEHK
GTIIGKQNDTCSTKTSCWLKSFKGFFYGLKNMLSGIFGNVFLGILLFLA
PFVLLIFFFFFGWRLLFCLKCCKKTKGLLKYKHLKDKEEAGYKKIIERLN
GKKGKSQLFDGERLANRKIAELFSTKIHIG

C-terminus

N-terminus

vLMs
MAEGGSSDCISRTQLVKTEVAEIHEDSYGGPGEKITICNGSTIVDQRL
GSELGCYTINRVKSYKLCENSASGKSCEIDSIPVKCKQGFCLKITQEGR
GHVKLSRGTEIVLDVCDSSCELMIPKGTGDILVDCSGGQQHFLQNNL
IDLGCPNVPLLGKMAIYVCRMSNHPKTTMAFLFWFSFGYVITCIVCR
VLFYLLIAIGTVGKKLKQYRELKPQTCIVCESVPVNAIDAEMHELNCSY
NICPYCASRLSTDGLIRHVTQCPKRREKVEETELFLNLERIPWLVRKLL
QVSESTGMALKRSCWMITLLLILLLVSMSPVQSAPVGKERAIETYQAR
EAYTGICLFVLGSVLFAVSWLTKALIDGIGNSFFPGLSVCKTCSIGSING
FEIESHKCYCSLFCCPYCRACSSDKNTHRMHLNVCKKRKMGSNVML
AVCKRMCFRATIEASNKALLIRSIINSTFVICILILTICVVSTSAVDMENL
PAGIWEKEEDLTNFCHQECQVTETECLCPYEAMVLRKPLFLDSIAKG
MKSLLNSTSLETSLSIEAPWGAINVQSTFKPTVSAANIALSWSSVEHR
GNKILVSGRSESIMKLEERTGVSWNLGVEDASESKTLTVSVMDLSQM
YSPVFEYLSGDRQVEEWPKATCTGDCPERCGCTSSTCLHKEWPHSR
NWRCNPTWCWGVGTGCTCCGLDVKDFFTDYMLVKWKVEYIKTEAI
VCVELTSQERQCSLIEAGTRFNLGPVTITLSEPRNVQQRLPPEIVTLHP
KIEEGFFDLMHVQKILSASTVCKLQSCTHGVPGDFQVYHVGNLLRG
DKVNGHSIHKIEPHFNTSWMSWDGCDLDYFCNMGDWPSCTYTGV
TQHNRAAFINMLNIETDYTKTFHFHSKRVTAHGDTPQLDLKARPAYG
AGEVTVLVEVADLELHTKKLEVSGLKIASLTCSGCYACSSGISCKVRIHV
NEPDEFTVHVRSSDPDVVAAGSSLMARKIEFGADSTFKAFSSMPKDS
LCFYIVEKDYCSSCTDDDTQKCVNTKLDHPQSILIEHKGTIIGKQNDTC
STKTSCWLKSFKGFFYGLKNMLSGIFGNVFLGILLFLAPFVLLIFFFFFG
WRLLFCLKCCKKTKGLLKYKHLKDKEEAGYKKIIERLNGKKGKSQLFD
GERLANRKIAELFSTKIHIG

C-terminus

Figure S1. Protein sequences CCHFV glycoprotein of antigens used in this study. Blue indicates the sequence for Gn. Orange indicates the sequence for Gc. Purple indicates the sequence for NSm.

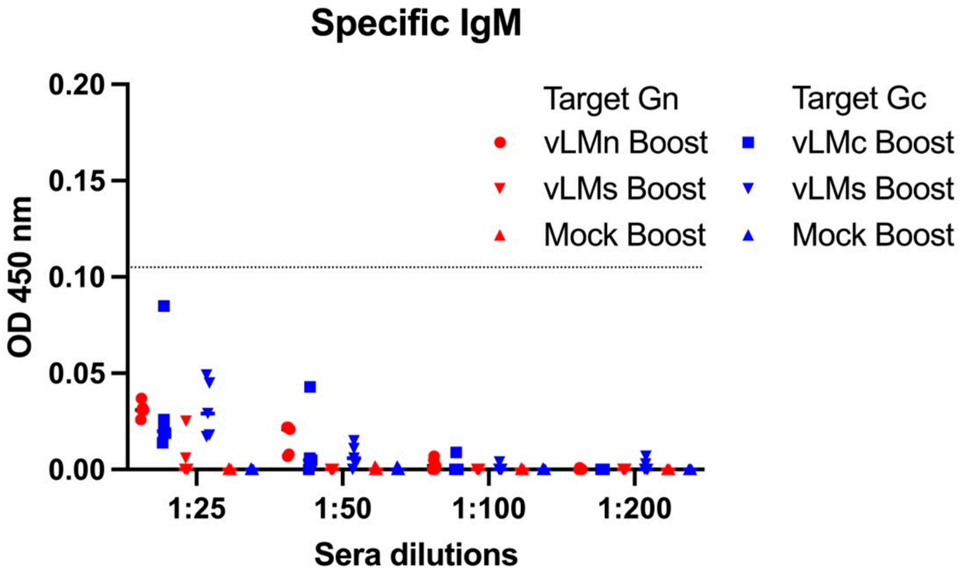

Figure S2. Specific IgM antibodies titer in C57BL/6J due to boost vaccination of mRNA vaccines. Sera were collected from the vLMn, vLMc, and vLMs vaccine groups after boost immunization, and the titer of IgM antibodies both targeting Gn and Gc were detected. The dotted lines represent the limit of detection.

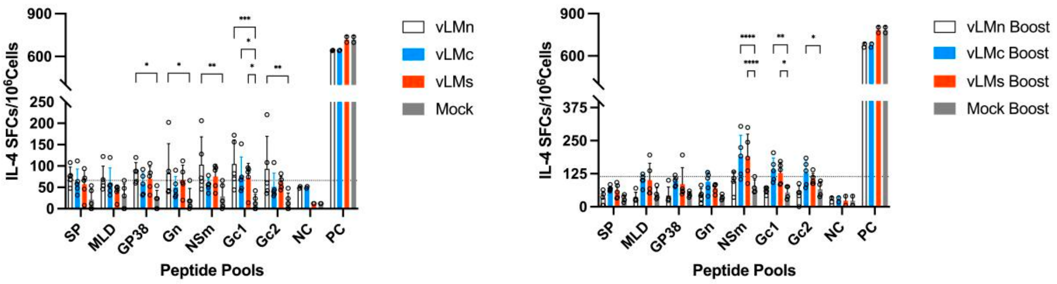

(**A**) (**B**)

Figure S3. IL-4 responses after prime and boost mRNA vaccinations. Fresh splenocytes from mice were stimulated with peptide pools including SP, MLD, GP38, Gn, NSm, Gc1 and Gc2 from GP of CCHFV YL16070 strain. ELISPOT was used to determine the number of IL-4 SFCs per 10^6 splenocytes (**A**) in primary and (**B**) boost vaccinations of vLMn, vLMc, vLMs and mock groups. Medium was used as the NC. PMA+Ionomycin was used as the PC. Dashed lines indicate the limit of detection. The symbols represent the individual animals in the groups. Data was shown as means ± SD, and was analyzed using two-way ANOVA with multiple comparison test. ****p<0.0001, ***p<0.001 *p<0.05.

Table S1. The sequences of UTR in mRNA vaccines.

Name	Sequence (5′ – 3′)
5′UTR	GAGAATAAACTAGTATTCTTCTGGTCCCCACAGACTCAGAGAGAACCCGCC<br>ACC
3′UTR	CAAGCACGCAGCAATGCAGCTCAAAACGCTTAGCCTAGCCACACCCCCACG<br>GGAAACAGCAGTGATTAACCTTTAGCAATAAACGAAAGTTTAACTAAGCTA<br>TACTAACCCCAGGGTTGGTCAATTTCGTGCCAGCCACACCCTGGTACTGCAT<br>GCACGCAATGCTAGCTGCCCCTTTCCCGTCCTGGGTACCCCGAGTCTCCCCC

	GACCTCGGGTCCCAGGTATGCTCCCACCTCCACCTGCCCCACTCACCACCTC TGCTAGTTCCAGACACCTCC
